# The atypical thiol–disulfide exchange protein α-DsbA2 from *Wolbachia pipientis* is a homotrimeric disulfide isomerase

**DOI:** 10.1107/S2059798318018442

**Published:** 2019-02-26

**Authors:** Patricia M. Walden, Andrew E. Whitten, Lakshmanane Premkumar, Maria A. Halili, Begoña Heras, Gordon J. King, Jennifer L. Martin

**Affiliations:** a Institute for Molecular Bioscience, University of Queensland, Brisbane, QLD 4072, Australia; b Griffith Institute for Drug Discovery, Griffith University, Nathan, QLD 4111, Australia

**Keywords:** DSB proteins, DsbA, *Wolbachia pipientis*, dithiol isomerases, dithiol oxidases

## Abstract

Disulfide-bond-forming (DSB) oxidative folding enzymes are master regulators of virulence that are localized to the periplasm of many Gram-negative bacteria. The archetypal DSB machinery from *Escherichia coli* K-12 consists of a dithiol-oxidizing redox-relay pair (DsbA/B), a disulfide-isomerizing redox-relay pair (DsbC/D) and the specialist reducing enzymes DsbE and DsbG that also interact with DsbD. By contrast, the Gram-negative bacterium *Wolbachia pipientis* encodes just three DSB enzymes. Two of these, α-DsbA1 and α-DsbB, form a redox-relay pair analogous to DsbA/B from *E. coli*. The third enzyme, α-DsbA2, incorporates a DsbA-like sequence but does not interact with α-DsbB. In comparison to other DsbA enzymes, α-DsbA2 has ∼50 extra N-terminal residues (excluding the signal peptide). The crystal structure of α-DsbA2ΔN, an N-terminally truncated form in which these ∼50 residues are removed, confirms the DsbA-like nature of this domain. However, α-DsbA2 does not have DsbA-like activity: it is structurally and functionally different as a consequence of its N-terminal residues. Firstly, α-DsbA2 is a powerful disulfide isomerase and a poor dithiol oxidase: *i.e.* its role is to shuffle rather than to introduce disulfide bonds. Moreover, small-angle X-ray scattering (SAXS) of α-DsbA2 reveals a homotrimeric arrangement that differs from those of the other characterized bacterial disulfide isomerases DsbC from *Escherichia coli* (homodimeric) and ScsC from *Proteus mirabilis* (PmScsC; homotrimeric with a shape-shifter peptide). α-DsbA2 lacks the shape-shifter motif and SAXS data suggest that it is less flexible than PmScsC. These results allow conclusions to be drawn about the factors that are required for functionally equivalent disulfide isomerase enzymatic activity across structurally diverse protein architectures.

## Abstract 2

The disulfide isomerase α-DsbA2 from *Wolbachia pipientisis* is unexpectedly homotrimeric and lacks the ‘shape-shifting’ conformational flexibility that defines another trimeric disulfide isomerase, ScsC from *Proteus mirabilis*.

## 1. Introduction   

Many secreted and outer membrane proteins of prokaryotes rely on disulfide bonds for their stability and function (Feige & Hendershot, 2011 ▸). The introduction, isomerization and reduction of protein disulfide bonds in bacteria are controlled by disulfide-bond-forming (DSB) proteins (Landeta *et al.*, 2018 ▸; Inaba, 2009 ▸). These DSB proteins are considered to be master regulators of virulence because they are essential for the folding and activity of diverse virulence factors, including bacterial toxins, secretion systems, adhesins, flagella *etc.* (Heras *et al.*, 2009 ▸).

The classic DSB folding machinery characterized in the model bacterium *Escherichia coli* K-12 comprises two independent periplasmic pathways: the (i) oxidative and (ii) isomerization pathways (Inaba, 2009 ▸). In the oxidative pathway, the monomeric thioredoxin (TRX)-fold protein *E. coli* DsbA (EcDsbA) donates its Cys-*X*-*X*-Cys active-site disulfide bond directly to nascent protein substrates (Zapun *et al.*, 1993 ▸; Inaba & Ito, 2002 ▸). EcDsbA becomes reduced as a consequence of this reaction and its active site is re-oxidized by a specific interaction with its integral membrane-protein partner *E. coli* DsbB (EcDsbB; Bader *et al.*, 1999 ▸).

In the classic isomerization pathway, the homodimeric protein disulfide isomerase *E. coli* DsbC (EcDsbC) reduces and shuffles incorrect disulfide bonds in misfolded proteins to generate correctly folded proteins (Shevchik *et al.*, 1994 ▸). Each EcDsbC protomer has a catalytic TRX-fold domain with a characteristic Cys-*X*-*X*-Cys active site, and an 87-residue N-terminal region that forms a dimerization domain that is essential for isomerase activity (McCarthy *et al.*, 2000 ▸). EcDsbC forms a redox relay with the integral membrane protein *E. coli* DsbD (EcDsbD) that maintains EcDsbC in its active reduced state (McCarthy *et al.*, 2000 ▸). *E. coli* K-12 also encodes two specialist reducing enzymes, EcDsbG and EcDsbE (Depuydt *et al.*, 2009 ▸), that interact with EcDsbD (Missiakas *et al.*, 1995 ▸)

Here, we focus on one of the DSB proteins encoded by *Wolbachia pipientis* *w*Mel, a bacterium from the Rickettsi­aceae family. The Rickettsiaceae family are Gram-negative bacteria of the α-Proteobacteria class that establish obligate intracellular infections in arthropods. *W. pipientis* is widespread, being found in ∼60% of insect species, and has an extraordinary impact on host biology. Infection results in phenotypic alterations such as cytoplasmic incompatibility, feminization or reduction of lifespan, all of which contribute to the survival of the bacterium (Hilgenboecker *et al.*, 2008 ▸). Several *Wolbachia* strains have been shown to block the transmission of mosquito-borne viruses and are being trialled as biocontrol agents aimed at eradicating vector-borne diseases such as Dengue, Zika and Chikungunya (reviewed in Flores & O’Neill, 2018 ▸).

The *W. pipientis* *w*Mel strain encodes two DsbA-like proteins, α-DsbA1 and α-DsbA2, and the integral membrane protein α-DsbB (Walden *et al.*, 2013 ▸). Unlike *E. coli*, this strain does not encode obvious homologues of DsbC or DsbD (although all other *W. pipientis* strains do encode a DsbD homologue). Of the two encoded DsbAs, α-DsbA1 has been characterized and shown to form a redox relay with α-DsbB (Walden *et al.*, 2013 ▸) that resembles the redox relay between EcDsbA and EcDsbB. In contrast, α-DsbA2, which is highly conserved in *Wolbachia*, does not interact with α-DsbB and has a long N-terminal region compared with α-DsbA1 (Walden *et al.*, 2013 ▸).

In EcDsbC, the N-terminal region forms a dimerization domain that is essential for disulfide isomerase activity. Two other DsbA-like proteins with N-terminal extensions are disulfide isomerases and are thought to be dimeric [*Legionella pneumophila* DsbA2 (LpDsbA2; Kpadeh *et al.*, 2015 ▸) and *Caulobacter crescentus* ScsC (CcScsC; Cho *et al.*, 2012 ▸)]. In addition, *Proteus mirabilis* ScsC (PmScsC) is DsbA-like with an N-terminal extension and has disulfide isomerase activity. However, its N-terminal residues interact to form a homotrimer (Furlong *et al.*, 2017 ▸). In each of these cases, the N-terminal regions are essential for oligomerization and protein disulfide isomerase activity. We hypothesized that the N-terminal region of *Wolbachia* α-DsbA2 would also impart disulfide isomerase activity by forming an oligomerization domain.

Here, we report structural and functional studies of *Wolbachia* α-DsbA2. We used two constructs: (i) FL α-DsbA2, the full-length mature α-DsbA2 (lacking the signal peptide that directs the protein to the periplasm) comprising residues 16–252, and (ii) α-DsbA2ΔN, a truncated form of α-DsbA2 (lacking both the signal sequence and the 50-residue N-terminal region) comprising residues 65–252. Our results show that FL α-DsbA2 is a strong protein disulfide isomerase and that the removal of the N-terminal residues eliminates this activity but gives rise to weak dithiol oxidase activity. The crystal structure of truncated α-DsbA2ΔN reveals a classic monomeric DsbA-like architecture. However, SAXS models of FL α-DsbA2 are consistent with a trimer forming through the interaction of the N-terminal residues.

## 2. Materials and methods   

### 2.1. Sequence analysis of α-DsbA2   

Analysis of the primary sequence was performed using the *ProtParam* proteomics server (Bairoch *et al.*, 2005 ▸) from ExPASy (Swiss Institute of Bioinformatics, Switzerland). The sequences of α-DsbA2, EcDsbC, PmScsC, CcScsC and LpDsbA2 were aligned in *ClustalOmega* (Sievers *et al.*, 2011 ▸) and secondary-structure predictions were carried out using *JPred* (Drozdetskiy *et al.*, 2015 ▸). The UniProt accession codes are as follows: EcDsbC, P0AEG6; PmScsC, B4EV21; CcScsC, Q9A747; α-DsbA2, Q73FL6; LpDsbA2, Q5WVK9. Structure-based sequence alignment of the catalytic domains of α-DsbA2, PmScsC and EcDsbC was determined with *PROMALS*3*D* (Pei *et al.*, 2008 ▸; the PDB coordinates used were 6eez, 4xvw and 1eej, respectively).

### 2.2. Protein expression and purification   

FL α-DsbA2 (locus WD1312; GenBank AE017196) was amplified from *W. pipientis* *w*Mel genomic DNA by PCR using the forward primer 5′-TAC TTC CAA TCC AAT GCG ATG AGC TTG CCG ATA ATA-3′ and the reverse primer 5′-TTA TCC ACT TCC AAT GCT AGC CTT GCT TGT GAC TTA A-3′, which incorporate overhangs for ligation-independent cloning (LIC). Truncated α-DsbA2ΔN was amplified using the forward primer 5′-TAC TTC CAA TCC AAT GCG GCT CGA GAT AAT GTA ACC-3′ and the reverse primer 5′-TTA TCC ACT TCC AAT GCT AGC CTT GCT TGT GAC TTA A-3′. The full-length mature protein without the signal peptide and α-DsbA2ΔN were cloned into the LIC vector pMCSG7 that incorporates a His_6_ tag, a linker region containing eight amino acids and a TEV protease cleavage site at the N-terminus of the inserted gene. The construct was transformed into the *E. coli* expression strain BL21(DE3)pLysS (Life Technologies, USA) to enable the overexpression of α-DsbA2 using autoinduction in ZYP-5052 medium at 30°C (Studier, 2005 ▸). The cells were collected using an Avanti J-25I centrifuge (Beckman Coulter, Australia) at 12 000*g* at 4°C for 10 min and were frozen at −80°C. α-DsbA2 variants were expressed and purified as described in Kurz *et al.* (2009 ▸) with minor variation in the lysis, wash and elution buffers. The lysis buffer consisted of 25 m*M* Tris–HCl pH 7.5, 150 m*M* NaCl; 25 m*M* imidazole was added for the washing buffer and 250 m*M* imidazole was included for protein elution.

### 2.3. Protein disulfide reductase assay   

The ability of FL α-DsbA2 and α-DsbA2ΔN to catalyse the reduction of insulin in the presence of DTT was measured *in vitro* (Holmgren, 1979 ▸). Insulin comprises two chains, A and B, which are linked via two disulfide bonds. Upon the reduction of the disulfide bonds by a high reductase-active catalyst, chain B becomes insoluble and precipitates. FL α-DsbA2, α-DsbA2ΔN, EcDsbC (positive control) or EcDsbA (negative control) were diluted to a final concentration of 10 µ*M* in buffer consisting of 100 m*M* sodium phosphate pH 7.0, 1 m*M* EDTA, 0.33 m*M* DTT. Insulin (at a final concentration of 0.13 m*M*) was added to the cuvette immediately before the measurements were taken, and the extent of insulin reduction was monitored by measuring the optical density at 650 nm for 50 min. Three experimental replicates were measured using the same batch of protein and the data presented data [mean ± standard deviation error (SD)] are from all three measurements.

### 2.4. Redox-potential measurement   

2 µ*M* oxidized FL α-DsbA2 and α-DsbA2ΔN was incubated in fully degassed buffers consisting of 100 m*M* sodium phosphate pH 7.0, 1 m*M* EDTA, 1 m*M* oxidized glutathione (GSSG; Sigma–Aldrich, USA) and different concentrations of reduced glutathione (GSH; 20 µ*M*–5 m*M*) for 24 h at room temperature. After incubation, the reactions were stopped with 10% trichloroacetic acid (TCA; Sigma–Aldrich, USA) and the precipitated protein pellets were collected by centrifugation at 16 000*g* for 10 min at 4°C. The pellets were washed with cold acetone and dissolved in buffer consisting of 50 m*M* Tris–HCl pH 7.0, 1% SDS, 4 m*M* 4-acetamide-4′-maleimidylstilbene-2,2′-disulfonate (AMS; Molecular Probes, USA). The reduced and oxidized forms were separated on a 12% SDS Bis–Tris PAGE (Invitrogen, Australia). The fraction of reduced protein, *R*, was determined from a scanned image of the stained gel using *ImageJ* (Abramoff *et al.*, 2004 ▸). The equilibrium constant *K*_eq_ was calculated via *R* = {[GSH]^2^/[GSSH])/(*K*_eq_ + ([GSH]^2^/[GSSH])}, and the redox potential was calculated via the Nernst equation *E* ^0^ = *E* ^0^_GSH/GSSG_ − (*RT*/*nF*)ln*K*_eq_, where *E* ^0^_GSH/GSSG_ = −240 mV, *R* = 8.314 J K^−1^ mol^−1^, *T* = 298 K, *n* = 2 and *F* = 9.649 × 10^4^ C mol^−1^ (Kurz *et al.*, 2009 ▸). Three experimental replicates were measured using different batches of protein and the data presented are the mean ± SD from duplicate measurements.

### 2.5. Dithiol oxidase activity assay   

Assays were run on a Synergy H1 Multi-Mode plate reader (BioTek, USA) with excitation at 340 nm and emission at 615 nm. For time-resolved fluorescence, a 100 µs delay before reading and a 200 µs reading time were employed. The assay was performed in a white 384-well plate (PerkinElmer). A 25 µl solution consisting of 3.2 µ*M* EcDsbA and 2 m*M* GSSG (positive control) or of 3 µ*M* FL α-DsbA2 or α-DsbA2ΔN and 2 m*M* GSSG in 50 m*M* MES, 50 m*M* NaCl, 2 m*M* EDTA pH 5.5 was added to the wells. The assay was initiated by the addition of 25 µl 16 µ*M* peptide (in 50 m*M* MES, 50 m*M* NaCl, 2 m*M* EDTA pH 5.5) to each well. The peptide substrate was CQQGFDGTQNSCK, with europium bound to a 1,4,7,10-tetraazacyclododecane-1,4,7,10-tetraacetic acid (DOTA) group amide-coupled to the N-terminus, and a methyl­coumarin amide-coupled to the ∊-amino group of the C-terminal lysine (AnaSpec, USA). Reconstitution of the peptide substrate solution is described in Walden *et al.* (2012 ▸). Measurements were carried out in triplicate using three different protein batches and the data shown are the mean ± SD error from these three measurements.

### 2.6. Protein disulfide isomerase assay   

The scrambled RNase A assay was used to detect the isomerase activity of FL α-DsbA2, α-DsbA2ΔN and EcDsbC by monitoring the refolding of scrambled RNase A (Hillson *et al.*, 1984 ▸). When the four randomly oxidized disulfides are correctly paired, the RNase is natively folded and active and can convert cyclic cytidine-3,5′-monophosphate (cCMP) into 3′CMP, which can be monitored colorimetrically. In these experiments, FL α-DsbA2, α-DsbA2ΔN or EcDsbC (10 µ*M* final concentration) was used in a buffer consisting of 100 m*M* sodium phosphate, 1 m*M* EDTA pH 7.0, 10 µ*M* dithiothreitol (DTT) and 40 µ*M* of scrambled RNase A. Scrambled RNase A was produced as described previously (Kurz *et al.*, 2009 ▸). At various time points, 50 µl of the reaction was mixed with 150 µl cytidine 3′,5′-cyclic monophosphate (3 m*M*) and the hydrolysis activity was monitored using a Biotek H1 plate reader (Millennium Science, USA) at 296 nm and 298 K. Native RNase A and scrambled RNase A samples without added enzyme served as positive and negative controls, respectively. Measurements were performed in triplicate using three different batches of protein and the data presented are the mean ± SD from three measurements.

### 2.7. Crystallization and crystal structure determination of α-DsbA2ΔN   

Crystal screening and optimization of α-DsbA2ΔN crystals was performed at the UQ ROCX facility (University of Queensland, Australia). For crystallization, the hanging-drop vapour-diffusion method was used. Crystals of α-DsbA2ΔN were grown by mixing 1 µl protein solution at 20 mg ml^−1^ and 1 µl crystallant solution consisting of 100 m*M* Tris pH 7.5, 200 m*M* NaCl, 20%(*w*/*v*) PEG 3350. X-ray data were recorded on the microcrystallography beamline MX2 at the Australian Synchrotron using the *Blu-Ice* software (McPhillips *et al.*, 2002 ▸). Reflections were processed in *XDS* (Kabsch, 2010 ▸), analysed and converted to MTZ in *SCALA* (Evans, 2006 ▸). Phases were obtained by molecular replacement using remote sequence homologues identified by *Fold and Function Assignment* (*FFAS*; Jaroszewski *et al.*, 2011 ▸). The top *FFAS* hit, *Salmonella enterica* serovar Typhimurium ScsC (SeScsC; PDB entry 4gxz; Shepherd *et al.*, 2013 ▸), shares 23% sequence identity with α-DsbA2ΔN. An initial search using the complete PDB coordinates of SeScsC as a model was unsuccessful. Instead, a trimmed polyserine template that retained the side chains of residues that were conserved in an alignment of SeScsC with α-DsbA2ΔN was used to phase the structure of α-DsbA2ΔN using *Phaser* (McCoy *et al.*, 2007 ▸). The root-mean-square deviation between these two structures is 1.8 Å for 124 equivalent C^α^ atoms. Further refinement was performed using *PHENIX* (Adams *et al.*, 2010 ▸, 2011 ▸) and *Coot*. Molecular figures were generated in *PyMOL* (v.1.2r3pre; Schrödinger). R.m.s.d. calculations and structural alignments were conducted using *PyMOL* as well as *DaliLite* (Holm *et al.*, 2008 ▸). The data-collection and refinement statistics are given in Table 1 ▸. The refined model of α-DsbA2ΔN has four molecules in the asymmetric unit, with chains *A*, *B*, *C* and *D* refined with 185, 189, 180 and 180 residues, respectively. Molecules *A* and *B* are both well defined in the electron density, but poor-density regions in molecule *C* (residues 188–197) and molecule *D* (residues 92–98 and 193–197) could not be improved during refinement (*e.g.* with only the C^α^ atoms refined and occupancies set to 0 for any other atoms in these regions). The different quality of these molecules is evident from the average *B* factors of chains *A* and *B* (46 and 41 Å^2^, respectively) and chains *C* and *D* (63 and 70 Å^2^, respectively) (see Fig. 3*b*). The catalytic disulfide bonds in molecules *A* and *B* were modelled in a mixed redox state (Fig. 3), whereas in molecules *C* and *D* they were modelled as reduced.

### 2.8. Electrostatic surface potential calculation and visualization of hydrophobicity surfaces   

The *Adaptive Poisson–Boltzmann Solver* (*APBS*) was used to calculate surface electrostatic potentials for α-DsbA2, PmScsC and EcDsbC using the nonlinear Poisson–Boltzmann equation (Baker *et al.*, 2001 ▸). The structures were superimposed and similarly oriented before the calculations were performed. We used the PARSE partial atomic charges and radii, internal and external dielectric constant values of 2 and 78, respectively, and solvent and ionic probe radii of 1.4 and 2 Å, respectively. Electrostatic potential calculations were performed with an ionic strength corresponding to a 150 m*M* monovalent counterion concentration at a temperature of 310 K.

To visualize the hydrophobic surface patches on α-DsbA2, PmScsC and EcDsbC (Fig. 4), we used *UCSF Chimera* (Pettersen *et al.*, 2004 ▸). Amino-acid residues were mapped to the hydrophobicity scale of Kyte & Doolittle (1982 ▸). In Fig. 4, the most polar residues are shown in purple and the most hydrophobic residues are shown in tan.

### 2.9. Small-angle X-ray scattering (SAXS) of FL α-DsbA2 and α-DsbA2ΔN   

SAXS data for α-DsbA2ΔN and FL α-DsbA2 were collected on the SAXS/WAXS beamline at the Australian Synchrotron (Kirby *et al.*, 2013 ▸). Data reduction was carried out using *scatterBrain* (v.2.71; Australian Synchrotron; http://archive.synchrotron.org.au/aussyncbeamlines/saxswaxs/software-saxswaxs) and the data were corrected for solvent scattering, sample transmission and detector sensitivity. For α-DsbA2ΔN, serial dilutions of an ∼5 mg ml^−1^ stock were loaded into a 96-well plate, while FL α-DsbA2 was measured using an inline SEC–SAXS setup (see Table 2 ▸). The estimated molecular mass was calculated using contrast and partial specific volumes determined from the protein sequences (Whitten *et al.*, 2008 ▸). Data processing and Guinier analysis was performed using *PRIMUS* (v.3.2; Konarev *et al.*, 2003 ▸). The pair-distance distribution function [*p*(*r*)] was generated from the experimental data using *GNOM* (v.4.6; Svergun, 1992 ▸), from which *I*(0), *R*_g_ and *D*_max_ were determined. *DAMMIN* (v.5.3; Svergun, 1999 ▸) was used to generate 16 dummy-atom models for each protein (assuming *C*_1_ point-group symmetry for α-DsbA2ΔN and *C*_3_ point-group symmetry for α-DsbA2ΔN), which were averaged using *DAMAVER* (v.2.8.0; Volkov & Svergun, 2003 ▸), and the resolutions of the averaged structures were estimated using *SASRES* (Tuukkanen *et al.*, 2016 ▸). All 16 dummy-atom models were used in the averaging procedure for α-DsbA2ΔN, but only nine (oblate) of the 16 dummy-atom models were averaged for FL α-DsbA2. Rigid-body modelling was carried out using *CORAL* (v.1.1; Franke *et al.*, 2017 ▸). For α-DsbA2ΔN, residues Asp68–Leu247 were taken from the crystal structure and treated as a rigid unit, while five additional residues were included at the N- and C-termini and treated as flexible linkers. For FL α-DsbA2, *C*_3_ symmetry was assumed and Leu17–Arg27 (a model helical segment generated by the three-dimensional modelling program *I-TASSER*; Zhang, 2008 ▸), Asp34–Glu57 (a model helical segment generated by *I-TASSER*) and Ala65–Leu247 (from the α-DsbA2ΔN crystal structure) were taken as rigid subunits, while five additional residues added at the N- and C-termini plus the intervening regions between the rigid segments were treated as flexible linkers. As oligomerization occurs through the N-terminal region and the model helices were amphipathic, Leu17, Ile20, Trp23, Ile36, Leu40, Ile44, Phe48, Val52 and Leu55 were restrained to be less than 15 Å from the same residue in a symmetry-related chain.

## 3. Results   

### 3.1. The N-terminus of *Wolbachia* α-DsbA2 is predicted to be helical   

**Figure 1:**
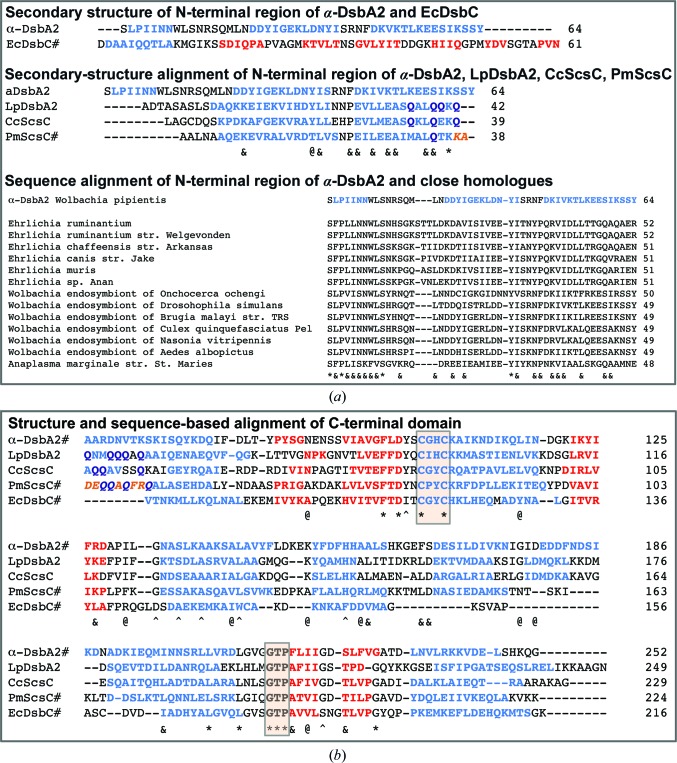
Comparison of the sequences and structures of bacterial disulfide isomerases. (*a*) Top panel: alignment of the N-terminal regions of α-DsbA2 (excluding the signal peptide residues 1–15) and EcDsbC. Secondary structure was determined from the structure of EcDsbC (PDB entry 1eej) or was predicted using *JPred* (Drozdetskiy *et al.*, 2015 ▸) for α-DsbA2. Blue, helices; red, strands. Middle panel: sequence alignment of the N-terminal regions of α-DsbA2, LpDsbA2, CcScsC and PmScsC. Secondary structure for α-DsbA2, LpDsbA2 and CcScsC was predicted by *JPred* and that for PmScsC was determined from the structure (PDB entry 4xvw). Blue, helices; red, strands; orange, the shape-shifter peptide that adopts different conformations in PmScsC (Furlong *et al.*, 2017 ▸), although predicted to be helical by *JPred*. Dark blue letters indicate glutamine residues in the shape-shifter region of all four proteins. In this alignment, one residue is conserved across the N-terminal regions of all four proteins and is marked ‘*’, one residue is conserved across α-DsbA2, LpDsbA2 and CcScsC and is marked ‘@’, and nine residues are conserved across LpDsbA2, CcScsC and PmScsC and are marked ‘&’. Bottom panel: sequence alignment of the N-terminal regions of α-DsbA2 and close homologues. The homologues were obtained using *BLASTp* (Altschul & Koonin, 1998 ▸) and were aligned according to sequence matching to α-DsbA2. In this alignment, fully conserved residues are marked ‘*’ and residues that are conserved in at least seven of the 13 α-DsbA2 homologues are marked ‘&’. (*b*) Structure- and sequence-based alignment of the C-terminal domains of α-DsbA2, LpDsbA2, CcScsC, PmScsC and EcDsbC. The secondary structures of α-DsbA2, PmScsC and EcDsbC are from their structures; the secondary structures of LpDsbA2 and CcScsC are those predicted by *JPred*. The C*XX*C active site and *cis*-proline loop residues are identified by shaded boxes. Sequence colour key: blue, helices; red, strands; orange italics, shape-shifter peptide; dark blue, glutamine residues in the shape-shifter peptide region. In this composite alignment, the ten residues that are conserved in all five proteins are marked ‘*’, the additional seven residues that are conserved across the four proteins α-DsbA2, LpDsbA2, CcScsC and PmScsC (but not EcDsbC) are marked ‘^’, the ten residues that are conserved across the three proteins α-DsbA2, LpDsbA2 and CcScsC (but not PmScsC) are marked ‘@’, and the seven residues that are conserved across LpDsbA2, CcScsC and PmScsC (but not α-DsbA2) are marked ‘&’. In all alignments, the sequences for which structures are known are marked ‘#’.

The 87-residue N-terminal region of the archetypal disulfide isomerase EcDsbC adopts a β-sheet dimerization domain with a helix that links to the catalytic domain (McCarthy *et al.*, 2000 ▸). The presence of a detectable DsbA-like domain and an extended N-terminal region in the *Wolbachia* α-DsbA2 sequence suggested that, like EcDsbC, the 50-residue N-terminal region might act as a dimerization domain. However, the predicted secondary structure of the α-DsbA2 N-terminal region has no structural relationship to the equivalent region of EcDsbC (Fig. 1 ▸ *a*).

Recently, three other DsbA-like proteins have been shown by gel filtration to be oligomeric as a consequence of their N-terminal residues; all three are functional disulfide isomerases. These are *L. pneumophila* DsbA2 (LpDsbA2; probably dimeric, with an ∼50-residue N-terminal region that is predicted to be helical; Kpadeh *et al.*, 2015 ▸), *C. crescentus* ScsC (CcScsC; probably dimeric, with an ∼60-residue N-terminal region that is predicted to be helical; Cho *et al.*, 2012 ▸) and *P. mirabilis* ScsC (PmScsC; a confirmed trimeric protein; ∼60-residue helical N-terminal region; Furlong *et al.*, 2017 ▸). An alignment based on the predicted or known secondary structures of these four proteins (α-DsbA2, LpDsbA2, CcScsC and PmScsC) is provided in Fig. 1 ▸(*a*), showing that they are all predicted to be helical, and these helical regions can be aligned. We know that PmScsC has a shape-shifting motif that is predicted to be helical but which can actually adopt helical, strand or coil structures (this region is shown in bold orange italic font in Figs. 1 ▸ *a* and 1 ▸ *b*). Interestingly, there is more in common across the sequences of LpDsbA2, CcScsC and PmScsC in this region than there is with α-DsbA2. This includes a preponderance of glutamine residues in the regions aligning in or near the shape-shifting region of PmScsC (Figs. 1 ▸ *a* and 1 ▸ *b*). Regions that are rich in glutamine are often associated with intrinsic disorder in proteins (Dyson & Wright, 2005 ▸). The middle panel of Fig. 1 ▸(*a*) also indicates that the sequences of LpDsbA2, CcScsC and PmScsC have ten conserved residues among the ∼40 residues in this N-terminal region, whereas α-DsbA2 has just two residues that are conserved in LpDsbA2 and CcScsC. Moreover, there are no glutamine residues in this region of α-DsbA2. The similarity in secondary-structure prediction and difference in glutamine enrichment suggests that like PmScsC, LpDsbA2 and CcScsC may have a shape-shifting region. However, α-DsbA2 appears to be the odd one out; its N-terminal region is different and is unlikely to be shape-shifting. Taken together, these data indicate that the N-terminal regions of LpDsbA2, CcScsC and PmScsC are similar to each other, and perhaps share the shape-shifting characteristics of PmScsC. However, this shape-shifting feature, and likely the dynamic motion associated with this motif, is likely to be absent in α-DsbA2 and its close homologues.

An alignment of the C-terminal domain of α-DsbA2 (comprising a thioredoxin fold and an inserted helical domain) with those of the other bacterial protein disulfide isomerases is shown in Fig. 1 ▸(*b*). This alignment suggests that α-DsbA2, LpDsbA2 and CcScsC are more similar to each other (four helices in the helical domain, the same number as in EcDsbA) than they are to PmScsC (which has three helices in the helical domain) or EcDsbC (which has two helices in the helical domain).

### 3.2. α-DsbA2 is redox-active and has disulfide isomerase activity as a consequence of its N-terminal residues   

**Figure 2:**
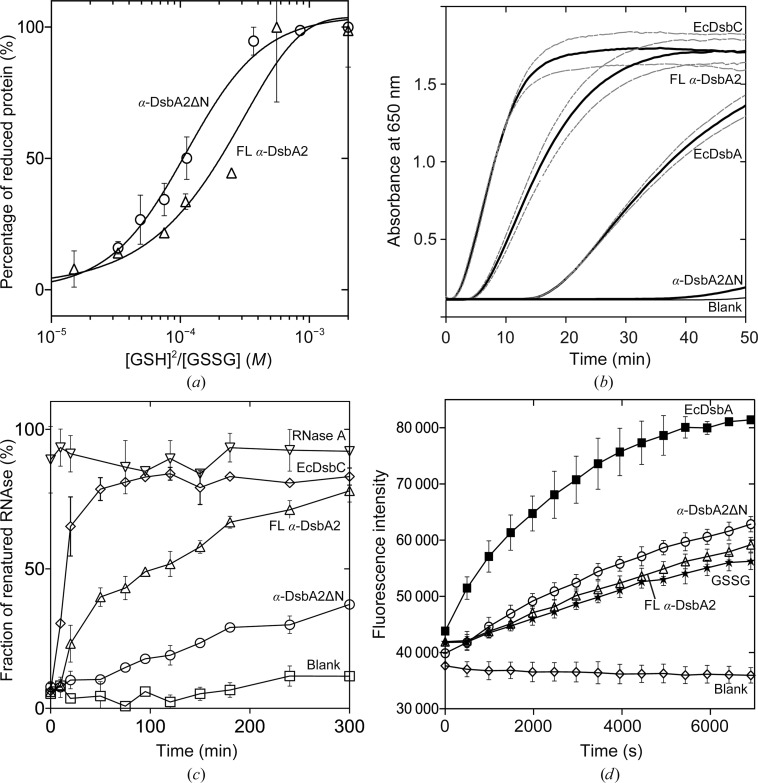
Redox properties of α-DsbA2. (*a*) Redox potential. The equilibria between reduced and oxidized FL α-DsbA2 (triangles) and α-DsbA2ΔN (circles) are shown. Data are presented as the mean ± SD of two measurements. (*b*) Protein disulfide reductase activity. An insulin-reduction assay was performed to measure the ability of α-DsbA2 variants to reduce insulin. The reduction of insulin, catalyzed by the protein or noncatalyzed (blank), was monitored at 650 nm over 50 min. The mean (black line) and standard deviation error (light grey lines) of three replicate measurements are shown. Dithiol oxidase (EcDsbA) and disulfide isomerase (EcDsbC) activities are shown for comparison. (*c*) Protein disulfide isomerase activity. Isomerization of scrambled RNase A in the presence of EcDsbC (diamonds), FL α-DsbA2 (triangles) or α-DsbA2ΔN (circles), positive control (RNase A; inverted triangles) and blank (scrambled RNase A without enzyme; squares). Data are presented as the mean ± SD of three replicate measurements. (*d*) Dithiol oxidase activity. Fluorescence curves showing dithiol oxidation (in the presence of GSSG) of a model peptide by EcDsbA (positive control; squares), α-­DsbA2ΔN (circles), FL α-DsbA2 (triangles), GSSG (without enzyme, *i.e.* negative control; stars) and α-DsbA2 without peptide (blank; diamonds). Data are presented as the mean ± SD of three replicate measurements.

We investigated the redox properties of FL α-DsbA2 and truncated α-DsbA2ΔN. The redox potential provides important information about the propensity of a protein to acquire electrons from its substrate and thereby become reduced. We determined the standard redox potentials of FL α-DsbA2 and α-DsbA2ΔN relative to the redox potential of glutathione (−240 mV; Fig. 2 ▸ *a*). From these data, the *K*_eq_ for FL α-DsbA2 was calculated to be 2.16 ± 0.15 × 10^−4^ *M*, corresponding to a redox potential of −131 mV at pH 7.0. The calculated *K*_eq_ for α-DsbA2ΔN was a little more oxidizing, 8.71 ± 0.12 × 10^−5^ *M*, corresponding to a redox potential of −122 mV. By comparison, the redox potential for *Wolbachia* α-DsbA1 is more reducing: *E* ^0^ = −163 mV (Kurz *et al.*, 2009 ▸). Thus, both the FL and truncated α-DsbA2 proteins have redox potentials that are similar to those of monomeric EcDsbA (*E* ^0^ = −122 mV; Mössner *et al.*, 1998 ▸) and dimeric EcDsbC (*E* ^0^ = −129 mV; Zapun *et al.*, 1995 ▸).

DSB enzymes are active to varying degrees in the standard disulfide reductase assay. We found that half of the insulin in solution was reduced by FL α-DsbA2 (which is in the reduced form owing to the DTT in the solution) after ∼15 min (Fig. 2 ▸ *b*). By comparison, EcDsbC reduced half of the insulin within ∼6 min and the oxidase EcDsbA (which is in the reduced form owing to the DTT in the solution) reduced half of the insulin after ∼35 min. The activity of α-DsbA2ΔN in this assay was negligible and was comparable to the negative control. These data show that FL α-DsbA2 is redox-active and that the N-terminal residues are critical for disulfide reductase activity.

We next assessed the protein disulfide isomerase activity of FL α-DsbA2 and α-DsbA2ΔN by following the reactivation of scrambled RNase A in an *in vitro* assay. FL α-DsbA2 recovered 73 ± 3% of the RNase A activity after ∼5 h, compared with the positive control EcDsbC which recovered 85 ± 5% of the RNase A activity over the same time period, although it reached this level after 100 min and then plateaued (Fig. 2 ▸ *c*). By comparison, truncated α-DsbA2ΔN recovered just 35 ± 3% of the RNase A activity relative to native refolded RNase A over this period, similar to that reported for the oxidase enzyme EcDsbA (Shouldice *et al.*, 2011 ▸). Therefore, we conclude that α-DsbA2 is a protein disulfide isomerase and that its disulfide isomerase activity requires the presence of the N-terminal residues.

We also investigated whether FL α-DsbA2 or α-DsbA2ΔN demonstrated dithiol oxidase activity by measuring their ability to catalyse disulfide-bond formation in an *in vitro* assay. Specifically, we measured the ability of FL α-DsbA2 or α-DsbA2ΔN to oxidize the cysteines of a model peptide in the presence of oxidized glutathione (GSSG). Compared with EcDsbA-mediated peptide oxidation, FL α-DsbA2 demonstrated negligible peptide oxidation activity (comparable to the GSSG control), whereas α-DsbA2ΔN had weak activity (Fig. 2 ▸ *d*) under the experimental conditions of this assay. EcDsbC is less active than EcDsbA and also has a lower activity than PmScsC in this assay (Furlong *et al.*, 2017 ▸).

### 3.3. The crystal structure of α-DsbA2ΔN reveals a canonical DsbA architecture   

**Figure 3:**
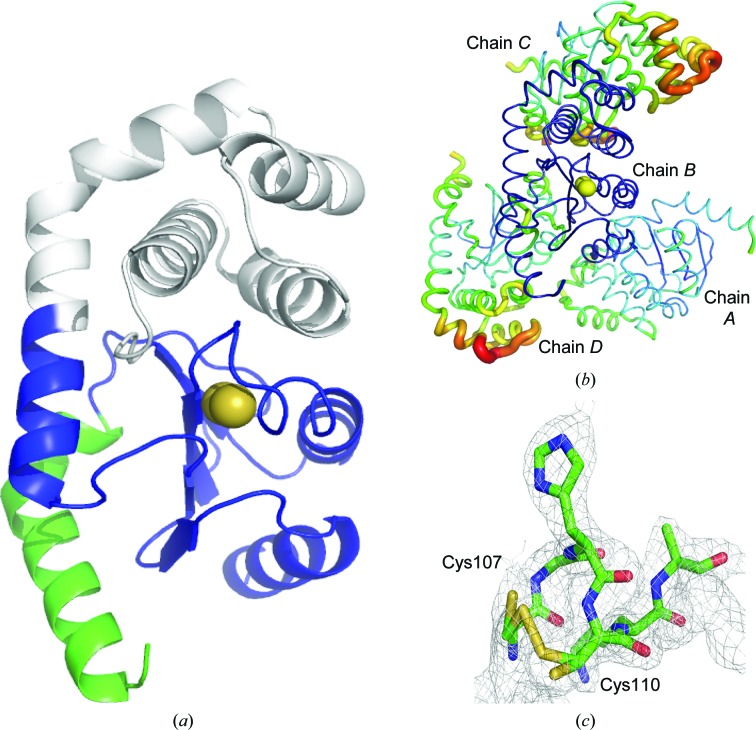
Structure of α-DsbA2ΔN. (*a*) Ribbon representation of α-DsbA2ΔN (chain *B* from the crystal structure) showing the thioredoxin domain in blue and catalytic cysteine S atoms as yellow spheres. The helical insertion is shown in grey and the N-terminus of the truncated protein is shown in light green. (*b*) The four molecules in the asymmetric unit. Chain *B* is oriented in the same way as in (*a*) and is shown in dark blue. The relative crystallographic temperature factors of the refined coordinates are shown by the backbone thickness (low to high shown as thin to thick) and for chains *A*, *C* and *D* by colour (low to high coloured from blue to red). (*c*) The active-site cysteines Cys107 and Cys110 of chain *B* were modelled in a mixed redox state (shown for chain *B*, with the 2*F*_o_ − *F*_c_ electron-density map contoured at 1.0σ).

We were unable to generate crystals of full-length mature α-DsbA2. However, crystals of the truncated α-DsbA2ΔN did grow and the crystal structure was solved by molecular replacement to a resolution of 2.25 Å (Table 1 ▸, Fig. 3 ▸ *a*).

The final refined structure of α-DsbA2ΔN has four molecules in the asymmetric unit (chains *A*, *B*, *C* and *D*; Fig. 3 ▸ *b*), each of which features the canonical DsbA architecture of a TRX fold with an inserted four-helical domain and a connecting helix. As in other DsbA-like crystal structures, the Cys-Gly-His-Cys active site is located at the N-terminal end of helix α3 in the TRX domain (present as a mixture of reduced and oxidized forms; Fig. 3 ▸ *a*).

Using this crystal structure as a probe, the highest-scoring *DALI* match (as of 1 March 2018) was the trimeric protein PmScsC (PDB entry 5idr, molecule *A*; Furlong *et al.*, 2017 ▸), with a *Z*-score of 25.8, an r.m.s.d. of 1.5 Å for 178 C^α^ atoms and 24% sequence identity. After PmScsC, the next highest *DALI* hit was an uncharacterized monomeric DsbA-like protein from *Silicibacter pomeroyi* (from the Rhodobacteraceae family; PDB entry 3gyk, molecule *A*; Midwest Center for Structural Genomics, unpublished work), with a *Z*-score of 24.8, an r.m.s.d. of 1.5 Å for 169 C^α^ atoms and 30% sequence identity. The third highest hit was the monomeric protein SeScsC (PDB entry 4gxz, molecule *C*; Shepherd *et al.*, 2013 ▸), with a *Z*-score of 24.1, an r.m.s.d. of 1.6 Å for 166 C^α^ atoms and 25% sequence identity (this was used as the molecular-replacement model to solve the crystal structure of α-DsbA2ΔN).

By comparison, superimposition of the α-DsbA2ΔN structure onto that of the archetypal disulfide isomerase EcDsbC (PDB entry 1eej, molecule *A*; McCarthy *et al.*, 2000 ▸) using *DALI* gave a *Z*-score of 13.5 and an r.m.s.d. of 3.3 Å for 147 C^α^ atoms (Maiti *et al.*, 2004 ▸). These data indicate that these two disulfide isomerases are structurally very different.

### 3.4. Surface features of α-DsbA2 reveal differences from other bacterial disulfide isomerases   

**Figure 4:**
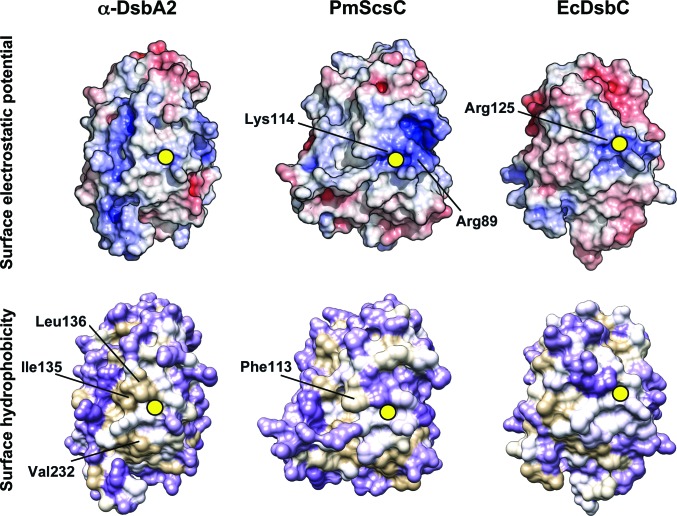
Comparison of surface properties. Electrostatic surface potentials of α-DsbA2, PmScsC and EcDsbC (top panel). The calculation of electrostatic surface potentials employed the nonlinear *Adaptive Poisson–Boltzmann Solver* (*APBS*) and the PARSE partial atomic charges and radii. Electrostatic surface potentials were contoured between −6 *kT* e^−1^ (red) and +6 *kT* e^−1^ (blue). The surface hydrophobicity of α-DsbA2, PmScsC and EcDsbC is presented in the bottom panel. The protein surface was mapped to the Kyte–Doolittle hydrophobicity scale from purple (most hydrophilic) to white to tan (most hydrophobic). The structures are arranged in a similar orientation to that of α-DsbA2 in Fig. 3 ▸(*a*). The position of the active-site cysteine is indicated by a yellow circle.

We generated the electrostatic surface potentials and the hydrophobicity surfaces for the catalytic domains of α-DsbA2 and two other structurally characterized disulfide isomerases: trimeric PmScsC and dimeric EcDsbC. These are compared in Fig. 4 ▸. Notably, there is a basic region near the catalytic site of both PmScsC and EcDsbC that is absent in α-DsbA2. This difference suggests different substrate preferences of the enzymes or possibly different contributions to redox properties. Fig. 4 ▸ also shows that the surface of α-DsbA2 near the catalytic site is largely hydrophobic. A hydrophobic region is also present in PmScsC and EcDsbC, and the conservation of this feature suggests this is a binding site for unfolded protein substrates.

### 3.5. SAXS shows that α-DsbA2 is a homotrimer as a consequence of its N-terminal region   

**Figure 5:**
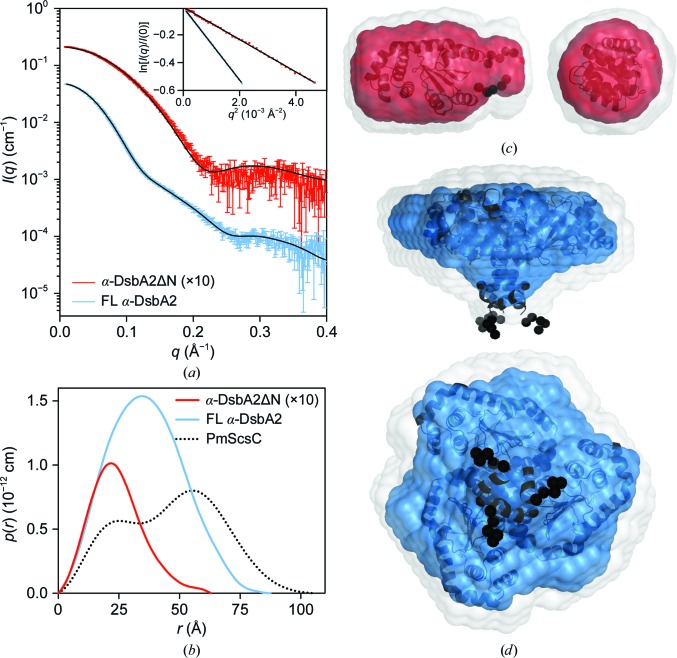
Small-angle X-ray scattering data for α-DsbA2ΔN and FL α-DsbA2. (*a*) Measured scattering data for α-DsbA2ΔN (red; multiplied by a factor of ten for clarity) and FL α-DsbA2 (blue). The scattering profiles of rigid-body models are shown as solid black lines overlaid on the scattering data for α-­DsbA2ΔN [χ^2^ = 3.88; CorMap test (Franke *et al.*, 2015 ▸), 294 points, *C* = 66, *P* = 0.000] and FL α-DsbA2 (χ^2^ = 1.37; CorMap test, 320 points, *C* = 13, *P* = 0.037). Inset: Guinier plot for α-DsbA2ΔN (red; *R* ^2^ = 0.999) and FL α-DsbA2 (blue; *R* ^2^ = 1.000). (*b*) The pair-distance distribution function, *p*(*r*), derived from the scattering data is indicative of a globular structure with a maximum dimension of ∼63 Å for α-DsbA2ΔN (red) and ∼87 Å for FL α-DsbA2 (blue). For reference, the experimental *p*(*r*) for trimeric PmScsC is also shown (dotted line). (*c*) Probable shape of α-DsbA2ΔN (monomeric) obtained from the filtered average of 16 dummy-atom models (red envelope): χ^2^ = 1.038 ± 0.002; NSD = 0.446 ± 0.021; resolution = 17 ± 2 Å. (*d*) Probable shape of FL α-DsbA2 obtained from the filtered average of nine dummy-atom models (blue envelope): χ^2^ = 1.202 ± 0.003; NSD = 0.602 ± 0.027; resolution = 30 ± 2 Å. The images in (*c*) and (*d*) were generated using *PyMOL* (v.1.2r3pre; Schrödinger), where the grey shapes represent the total volume encompassed by the aligned dummy-atom models and the corresponding rigid-body model is shown aligned with the filtered model (flexible regions are represented by chains of black spheres).

Although we were unable to crystallize the full-length protein, we were able to obtain low-resolution structural information using SAXS data from both FL α-DsbA2 and α-DsbA2ΔN (Table 2 ▸). Guinier plots (inset in Fig. 5 ▸ *a*) reveal a linear trend, consistent with both samples being monodisperse.

The bacterial disulfide isomerases characterized to date have been reported to be either dimeric (EcDsbC, CcScsC and LpDsbA2) or trimeric (PmScsC). We were therefore interested to determine the molecular mass of the full-length protein and identify whether it is dimeric or trimeric. Using SAXS data, the molecular mass of truncated α-DsbA2ΔN was estimated to be ∼23 kDa from *I*(0) (Orthaber *et al.*, 2000 ▸) and the Porod volume (Fischer *et al.*, 2010 ▸), which is very close to the expected mass for an α-DsbA2ΔN monomer (21 kDa) and is consistent with the crystal structure that we report here. However, the molecular masses of FL α-DsbA2 estimated from *I*(0) and the Porod volume (75 and 87 kDa, respectively) are consistent with the mass of a homotrimer (81 kDa) rather than a homodimer (54 kDa).

The *p*(*r*) for FL α-DsbA2 shows a single peak with a maximum dimension of ∼87 Å (Fig. 5 ▸ *b*), whereas α-DsbA2ΔN demonstrates a single peak with a significantly smaller maximum dimension of 63 Å. The larger dimension and shifting of the position of the peak in the *p*(*r*) for FL α-DsbA2 are consistent with the formation of a higher-order oligomer. While these data indicate that the FL α-DsbA2 oligomer is trimeric, this homotrimer differs from that of PmScsC (Furlong *et al.*, 2017 ▸), which has a bimodal pair-distance distribution function (dotted line in Fig. 5 ▸ *b*).

Both dummy-atom and rigid-body modelling were used to determine low-resolution solution structures of FL α-DsbA2 and α-DsbA2ΔN. The averaged and filtered dummy-atom model of α-DsbA2ΔN shows very good agreement with the α-DsbA2ΔN rigid-body model (composed of the crystal structure plus missing residues at the N- and C-termini; Fig. 5 ▸ *c*). A comparison of the scattering data with the rigid-body model scattering profile (red curve and black line in Fig. 5 ▸ *a*) shows good correspondence, although with a small systematic difference between the two curves that could be indicative of a low level of sample impurity or possibly a small difference between the solution and crystal structures.

The averaged and filtered dummy-atom model of FL α-DsbA2 reveals a disc-like structure with a small protrusion at the centre. Alignment of this model with the FL α-DsbA2 rigid-body model (Fig. 5 ▸ *d*) shows excellent correspondence; the N-terminal oligomerization domain of the rigid-body model coincides with the protrusion in the dummy-atom model, and the catalytic domains are positioned around the main disc. The scattering data for FL α-DsbA2 show excellent correspondence with the rigid-body model scattering profile (blue data points and black line, respectively, in Fig. 5 ▸ *a*).

**Figure 6:**
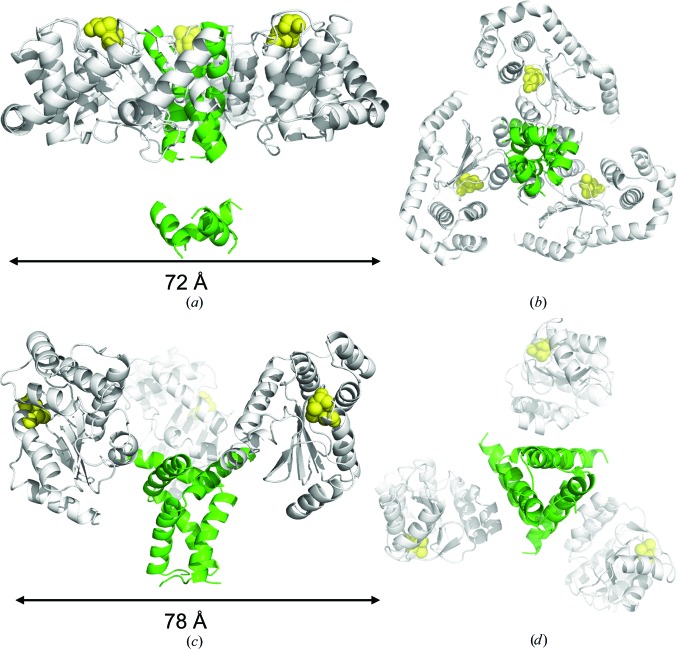
Comparison of rigid-body models of FL α-DsbA2 and PmScsC. Model of FL α-DsbA2 from (*a*) side and (*b*) bottom views. Model of PmScsC (SASBDB code SASDB94) from (*c*) side and (*d*) bottom views. For both FL α-DsbA2 and PmScsC the N-terminal trimerization domain is shown as a green ribbon, the catalytic domain as a white ribbon and the active-site residues as yellow spheres. The regions treated as flexible linkers in the rigid-body model are not shown, which is the reason for the apparent gap in the N-terminal trimerization domain shown in (*a*).

The two homotrimeric disulfide isomerase enzymes *Wolbachia* FL α-DsbA2 and PmScsC nevertheless have distinct solution structures. FL α-DsbA2 is disc-like with the three protomers tightly arranged into a compact shape (Figs. 6 ▸ *a* and 6 ▸ *b*), whereas PmScsC has a more open arrangement (Figs. 6 ▸ *c* and 6 ▸ *d*; Furlong *et al.*, 2017 ▸).

## 4. Discussion   

The *w*Mel strain of *W. pipientis* encodes two DsbA-like proteins. One of these, α-DsbA1, has previously been shown to be functionally similar to EcDsbA: it catalyses disulfide formation and forms a redox pair with a membrane-protein partner, α-DsbB (Walden *et al.*, 2013 ▸). Despite sequence similarity, the second protein, α-DsbA2, is not DsbA-like. It does not catalyse disulfide formation in the standard assay (this work) and it does not interact with α-DsbB (Walden *et al.*, 2013 ▸).

The unusually long N-terminus of *W. pipientis* *w*Mel α-DsbA2 is conserved in DsbA2s encoded by bacterial species from the family Rickettsiaceae in the class Alphaproteobacteria. This includes other members from the same class, including *Ehrlichia* and *Anaplasma*, that live in hosts such as ticks and cause diseases in animals and humans (Wormser *et al.*, 2006 ▸). These organisms all encode α-DsbA1 and α-DsbA2 enzymes similar to those encoded by *Wolbachia*. The high degree of conservation of the N-terminal residues in α-DsbA2 (bottom panel in Fig. 1 ▸ *a*) suggests that this region has an important function. Here, we have shown that these N-terminal residues confer trimerization and disulfide isomerase properties to *W. pipientis* *w*Mel α-DsbA2.

The enzymatic profile of *W. pipientis* *w*Mel α-DsbA2 overlaps with that of EcDsbC. Indeed, it may replace EcDsbC functionally since *Wolbachia* strains do not encode a DsbC. Most *Wolbachia* strains encode an EcDsbD-like protein that could act as a redox partner for α-DsbA2. Curiously, a DsbD homologue is not present in the genome of the specific *w*Mel *Wolbachia* strain that we investigated. We cannot explain why this might be or what other protein might serve as a reducing partner in this organism.

Other organisms encode a disulfide isomerase like α-DsbA2 from *Wolbachia* rather than EcDsbC. For example, *L. pneumophilia* does not contain a DsbC homologue (Kpadeh *et al.*, 2013 ▸, 2015 ▸), although like *Wolbachia* it has two DsbA-like proteins, one of which, LpDsbA2, has disulfide isomerase activity. LpDsbA2 has a predicted helical N-terminal extension (Fig. 1 ▸ *b*) and is essential for the assembly of the type 4b Dot/Icm secretion system (Kpadeh *et al.*, 2013 ▸, 2015 ▸). Curiously, the sequence of the N-terminal region of LpDsbA2 has more similarity to that of PmScsC than to that of α-DsbA2, suggesting that its structure and dynamic properties are more like those of PmScsC.

We have shown that *W. pipientis* *w*Mel α-DsbA2 is homotrimeric and has disulfide isomerase activity. This is the second reported example of a homotrimeric TRX-fold disulfide isomerase, with the other being PmScsC (Furlong *et al.*, 2017 ▸), although these two enzymes are quite distinct. Firstly, α-DsbA2 is structurally different from PmScsC in solution. PmScsC (Furlong *et al.*, 2017 ▸) exhibits a bimodal pair-distance distribution function, whereas FL α-DsbA2 has a single peak, indicating a more globular and compact shape. Both dummy-atom and rigid-body modelling reveal that FL α-DsbA2 is disc-like, with no evidence of the flexibility observed for PmScsC (Furlong *et al.*, 2017 ▸). Moreover, PmScsC is part of a highly conserved four-gene *scs* cluster that is associated with bacterial copper resistance, including its redox partner PmScsB (Furlong *et al.*, 2018 ▸), whereas α-DsbA2 is not part of a gene cluster. Finally, PmScsC is encoded in organisms that also encode DsbC-like enzymes, whereas α-DsbA2 is not. Presumably, PmScsC may play a specific role – perhaps it has a specific substrate associated with copper sensitivity – whereas α-DsbA2 may not.

Although these three structurally characterized disulfide isomerases, EcDsbC, PmScsC and α-DsbA2, differ considerably in their structures, we can draw some broad conclusions about the factors that contribute to their functionally equivalent enzymatic activities. The present work supports the notion that strong disulfide isomerase activity requires the presence of at least two catalytic domains in the enzyme. The way that these domains are brought together can vary (dimer/trimer or sheet/helix) and there is some limited variation in the catalytic active-site motif: CGYC in DsbC and PmScsC, CPYC in CcScsC, CIHC in LpDsbA2 and CGHC in *Wolbachia* α-DsbA2. Thus, glycine or proline predominate in the Cys+1 position and tyrosine or histidine predominate in the Cys+2 position. However, this catalytic motif sequence overlaps with that of monomeric dithiol oxidase DsbAs (for example CPHC in EcDsbA). The second motif that is highly conserved in TRX-like proteins is the *cis*-proline motif. Surprisingly, in all five of these disulfide isomerase enzymes the sequence motif is the same: GT*c*P. Monomeric DsbA-like oxidases tend to have more variation, with the glycine being highly variable and the threonine often replaced by valine. The most telling sequence feature that discriminates between oxidase and isomerase activity seems to be the addition of 50 or more residues at the N-terminus of the TRX fold that can form an oligomerization domain that can be either dimeric or trimeric and can sometimes contain a shape-shifter peptide.

## Supplementary Material

PDB reference: α-DsbA2, 6eez

## Figures and Tables

**Table 1:** X-ray data-collection and refinement statistics for α-DsbA2ΔN Values in parentheses are for the highest resolution shell.

Data collection	
Wavelength (Å)	0.9537
Resolution range (Å)	19.77–2.25 (2.32–2.25)
Space group	*P*2_1_
*a*, *b*, *c* (Å)	54.4, 104.8, 67.7
α, β, γ (°)	90.0, 96.5, 90.0
No. of molecules in asymmetric unit	4
Observed reflections	132099
Unique reflections	35423 (3209)
*R*_merge_	0.051 (0.265)
*R*_p.i.m._	0.038 (0.187)
Completeness (%)	99.04 (97.8)
〈*I*/σ(*I*)〉	13.6 (3.9)
Multiplicity	3.8 (3.6)
Refinement statistics	
*R* factor (%)	22.7 (20.9)
*R*_free_ (%)	27.8 (25.3)
Unique reflections	35423
No. of non-H atoms	
Total	6064
Protein	5799
Water	265
Average *B* factor (Å^2^)	58.0
R.m.s.d. from ideal geometry	
Bonds (Å)	0.002
Angles (°)	0.447
*MolProbity* analysis	
Ramachandran favoured/outliers (%)	97.12/0.0
Clashscore all atoms [percentile]	3.38 [99th]
*MolProbity* score [percentile]	1.36 [99th]

**Table 2:** Details of SAXS data collection and analysis

	α-DsbA2ΔN	FL α-DsbA2
Data-collection parameters		
Instrument	SAXS/WAXS (Australian Synchrotron)	
Beam geometry	Point	
Wavelength (Å)	1.033	1.127
Sample-to-detector distance (m)	1.575	1.575
*q*-range (Å^−1^)	0.01–0.57	0.01–0.48
Exposure time (s)	16 (8 × 2 s exposures)	40 (8 × 5 s exposures)
Configuation	Concentration series from 96-well plate	WTC-030S5 SEC–SAXS column: flow, 0.5 ml min; inject, 100 µl, 19.8 mg ml^−1^
Protein concentration range (mg ml^−1^)	1.25–5.00	0.35–2.20
Temperature (K)	283	293
Absolute intensity calibration	Water	
Sample details		
Extinction coefficient [*A*_280_, 0.1%(*w*/*v*)]	0.494	0.762
Partial specific volume (cm^3^ g^−1^)	0.735	0.735
Contrast, Δρ (10^10^ cm^−2^)	2.88	2.89
Molecular mass (from sequence) (kDa)	21.1	27.1
Protein concentration† (mg ml^−1^)	1.25	0.75
Structural parameters		
*I*(0) (from Guinier) (cm^−1^)	0.0215 ± 0.0001	0.04846 ± 0.00003
*R*_g_ (from Guinier) (Å)	18.7 ± 0.1	28.0 ± 0.1
*I*(0) [from *p*(*r*)] (cm^−1^)	0.0215 ± 0.0001	0.04845 ± 0.00002
*R*_g_ [from *p*(*r*)] (Å)	18.8 ± 0.1	27.9 ± 0.1
*D*_max_ (Å)	63 ± 3	87 ± 3
Porod volume (Å^3^)	27500 ± 1500	91300 ± 4500
Molecular-mass determination		
Molecular mass [from *I*(0)] (kDa)	23 ± 1	87 ± 5
Molecular mass (from Porod) (kDa)	23 ± 1	75 ± 5

† α-DsbA2ΔN: *I*(0) = 0.0427 ± 0.0001 cm^−1^, *R*_g_ = 18.9 ± 0.1 Å, *M* = 23 kDa (2.5 mg ml^−1^); *I*(0) = 0.0890 ± 0.0002 cm^−1^, *R*_g_ = 19.2 ± 0.1 Å, *M* = 24 kDa (5.0 mg ml^−1^). There is a small but significant systematic change in *R*_g_ and *M* in the concentration range measured, but based on the trend a concentration of 1.25 mg ml^−1^ was deemed to be free of concentration-dependent effects. FL α-DsbA2: at the peak concentration of ∼2.2 mg ml^−1^, *R*_g_ = ∼27.6 Å, which increases and plateaus at *R*_g_ = ∼28.0 Å at a concentration of between 0.35 and 1.15 mg ml^−1^. This range was deemed to be free of concentration-dependent effects and all eight frames collected over this concentration range were combined, where the average concentration was 0.75 mg ml^−1^.
